# Generation of Multi-Lobe Chua Corsage Memristor and Its Neural Oscillation

**DOI:** 10.3390/mi13081330

**Published:** 2022-08-17

**Authors:** Yue Liu, Hui Li, Shu-Xu Guo, Herbert Ho-Ching Iu

**Affiliations:** 1 Department of Electrical and Electronic Engineering, Changchun University of Technology, Changchun 130012, China; 2 College of Electronic Science and Engineering, Jilin University, Changchun 130012, China; 3 Department of Electrical, Electronic and Computer Engineering, The University of Western Australia, Crawley, WA 6009, Australia

**Keywords:** chua corsage memristor, small-signal equivalent circuit, edge of chaos, neural oscillation

## Abstract

The Chua corsage memristor (CCM) is considered as one of the candidates for the realization of biological neuron models due to its rich neuromorphic behaviors. In this paper, a universal model for *m*-lobe CCM memristor is proposed. Moreover, a novel small-signal equivalent circuit with one capacitor is derived based on the proposed model to determine the edge of chaos and obtain the zero-pole diagrams and analyze the frequency response and oscillation mechanism of the *m*-lobe CCM system, which are discussed in detail. In view of existence of the edge of chaos, the frequency response and the oscillation mechanism of the simplest oscillator is analysed using the proposed model. Finally, the proposed model has exhibited some essential neural oscillation, including the stable limit cycle, supercritical Hopf bifurcation, spiking and bursting oscillation. This study also reveals a previously undiscovered behavior of bursting oscillation in a CCM system.

## 1. Introduction

The development and complexity analysis of biological emotion-/memory-like models, especially the neural oscillation and design of the related mimicking electronic units, have become the hot topics in the fields of neuromorphic engineering and neuroergonomics. Due to their nature of unique memory, the memristors and memristive devices have become one of the strong candidates to achieve, simultaneously, scalability and biological flexibility, which push forward the next generation of neuromorphic computing. Therefore, as one of the general memristors, Chua corsage memristor (CCM) has been considered the key element to imitate the nonlinear biological behaviors and construct the neural network [1]. In 2016, the small-signal equivalent circuit and the simplest electronic oscillator consisting of only one CCM in parallel with a battery was presented [2]. Subsequently, a series of studies was conducted to investigate the complex characteristics of CCMs, such as Hopf bifurcation [2], the locally active [3,4], the edge of chaos [5], pinched hysteresis loops [6,7], basin of attraction [8,9], phase portraits basin of attraction, phase portraits [10], etc.

Hereby, some conclusions in the existing research literature could be reviewed as follows: (i) the theoretical studies and dynamics analysis on 2-/4-/6-lobe CCM systems [7,11,12,13,14] have been implemented by H. Kim and his team. Subsequently, the global dynamics, locally active, phase portraits, basin of attraction, pinched hysteresis loops, switching kinetics, physical realization, oscillation, and their emulator circuits [15,16] were analyzed. However, most of these research focused on several specific CCM systems. When the more lobes are needed, some important questions might be asked. For example, whether these typical nonlinear behaviors still occur in the other family of CCM, such as 12- or 14-lobe, even 20-lobe ones. What are the differences between commonality and individuality for different *m*-lobe CCMs? Since the small-signal equivalent circuit and the admittance function with one inductor has been designed in 2015, has other types of equivalent circuits with one capacitor and their impedance functions been developed and considered? Whether more than one similar oscillator could be built; (ii) the neural oscillation phenomenon have been found in some memristors and memristive systems, such as memristive synaptics, memristor-based neurons [17,18,19,20] and neural networks [21,22,23,24]. Then, the following studies should be worth pondering: whether most of existing models could be constructed based on the *m*-lobe CCM? When the biological neurons and neural networks needed to be simulated, why the *m*-lobe can be thought one of the powerful candidates? (iii) the applications, design, and physical implement of the CCM have also been carried out, such as the memristive self-learning logic circuit [25], in-memory computing [26], bistable nonvolatile [27], tri-stable locally-active, neuromorphic dynamics [28], neural oscillation dynamics [29,30], etc., but still in their infancy.

Based on the above literature review, the following topics with the aims of furthering research on the CCM are presented in this paper: (i) the universal model of the *m*-lobe CCM is introduced, which is helpful to answer the differences between commonality and individuality for all the families of CCMs; (ii) in order to investigate the versatility and flexibility for the proposed model, universal impedance function, and a novel small-signal equivalent circuit with one capacitor is designed, which is helpful for understanding the electronic circuit theory and analyzing dynamics behaviors; (iii) based on the existing theory, the nonlinear dynamics and neural oscillation on the proposed model are illustrated, such as the edge chaos, neural oscillation (i.e., supercritical Hopf bifurcation, spiking and bursting oscillation.), nonvolatile, coexisting pinched hysteresis loops, etc.

The remainder of this paper is organized as follows: in Section 2, the universal model of *m*-lobe Chua corsage memristor is introduced. We also summarize several natural characteristics of the proposed model, such as pinched hysteresis loops, DC V−I curves, and multiple locally-active domains. In Section 3, the universal impedance function is derived. Then, the novel small-signal equivalent circuit with a single capacitor is designed. The existence of the edge of chaos, zero-pole diagrams, and frequency response are discussed and summarized. Moreover, in Section 4, the distributed rules on the external positive inductance are desmonstrated. The oscillation mechanism, supercritical Hopf bifurcation, limit cycle, spiking and bursting oscillation of the simplest oscillator circuit is demonstrated based on the relationship between the model parameters and the excitation voltage. Finally, the paper is summarized in Section 5.

## 2. The Universal Model of *m*-Lobe Chua Corsage Memristor

The universal model of *m*-lobe CCM can be introduced as follows:

(1) $$\begin{array}{c} \left\{\begin{array}{c} \begin{array}{cc} i=G\left(x\right)v, & G\left(x\right)=G_{0}x^{2} \end{array}\\ \frac{dx}{dt}=A-x+\sum_{j=1}^{N}\left(\left|x-n_{j}\right|-\left|x-h_{j}\right|\right)+v\overset{\Delta }{\;=}f_{m}\left(x,v\right) \end{array}\right.\\ where\;A=\left\{\begin{array}{cc} A_{FS} & A_{FS}<A_{M}\\ A_{M}=\frac{1}{m}\sum_{j=1}^{N}\left(n_{j}+h_{j}\right)+a & A=A_{M}\\ A_{FL} & A_{FL}>A_{M}. \end{array}\right. \end{array}$$

**Figure 1:**
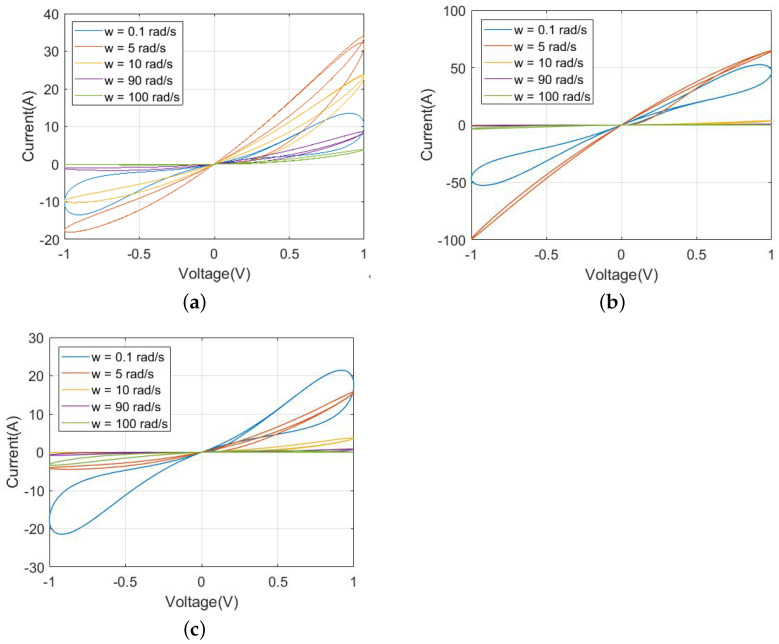
The coexisting pinched hysteresis loop: (**a**) $A=A_{M}$; (**b**) $A=A_{FS}<A_{M}$; (**c**) $A=A_{FL}>A_{M}$.

G(x) is the memductance function and $G_{0}$ ($G_{0}=0.1$) is a real constant; *i*, *v* and *x* are denoted as the current, voltage and state variable of the CCM, respectively. *A* and $a={\left(N-1\right)}^{2}+1$, (*N* is an integer, N=2,3,4,…) are the real parameters; $A_{M}$ is called the midpoint briefly; both $n_{j}$ and $h_{j}$ are the appropriate integers ($h_{j}>n_{j}$); m=2,4,6,… represents the number of the lobe. Then, the coexisting pinched hysteresis loops are shown in Figure 1.

**Proposition** **1.** *For the proposed universal model, the parameter A exhibits three cases, which imply the quite different conditions and negative slope domains (namely, locally active regions), the related statement as follows:*

*Case 1: When the parameter $A=A_{M}$ is fixed, only one negative slope domain exists in the first stable branch, which is located over the negative range of the voltage;*

*Case 2: When the parameter $A=A_{FS}<A_{M}$ is chosen, only one negative slope domain could be observed in the first branch, which is located over the positive range of the voltage;*

*Case 3: When the parameter $A=A_{FL}>A_{M}$ is obtained, two the negative slope domains could be obtained in the first and second stable branch.*

**Proposition** **2.** *Several natural characteristics of the proposed models in both v vs. i and dx/dtvs. x are shown as follows:*

*(1) m+1 equilibrium ($i.e.,Q_{0},Q_{1},Q_{2},\ldots ,Q_{m}$) and m turning points (namely., $T_{1},T_{2},T_{3},\ldots ,$$T_{m}$) could be observed in their dynamic route maps (DRMs);*

*(2) Substitute the x-axis coordinate of the first DC operating point ($T_{1}$) into f(x), then $f(T_{1})<0$;*

*(3) Substitute the x-axis coordinate of the last DC operating point ($T_{m}$) into f(x), then $f(T_{m})>0$;*

*(4) The properties and nonlinear phenomena (i.e., the location of DC operating points, local stability, active and locally active regions, oscillation, etc.) would not disappear as the number of lobes increase;*

*(5) Edge of chaos can occur in the first lobe, but do not change and disappear as the number of lobes increase;*

*(6) Similar neural oscillation dynamical behaviors might emerge. However, they are not identical phenomena in all the m-lobe CCMs.*

*Notably*, before analyzing the proposed universal CCM model, some statements need to be elaborated:

(i) the above features (4)–(6) would be verified via Figure 2, Figure 3, Figure 4, Figure 5, Figure 6 and Figure 7. Observed from these Figure 3, Figure 4, Figure 5, Figure 6, Figure 7, Figure 8 and Figure 9, the negative slopes are mainly concentrated in the first lobe;

(ii) there are plenty of ways to set the values of the parameters $n_{j}$ and $h_{j}$. In this paper, one method is chosen to obtain the *m*-lobe CCM, i.e., $n_{j}=\left(j+1\right)\left(j+2\right)$, $h_{j}=n_{j+1}$, (j=1,2,…);

(iii) since the complexity and dynamics oscillation are mainly concentrated in the first lobe, a 2-lobe CCM as an example can be considered an example, the verification and discussion on the above characteristics are given as follows.

When driven by one periodic input voltage/current excitation with a zero DC component, the frequency-dependent pinched hysteresis loops become a signature. Next, the Lissajous figures of the proposed model in the i−v plane with a sinusoidal input signal are exhibited in Figure 1.

As expected, from Figure 1, the pinched hysteresis loop for amplitude, $A_{m}$ = 1, exists for all frequencies, ω = 0.1 rad/s, 5 rad/s, 10 rad/s, 90 rad/s, and 100 rad/s. All pinched hysteresis loops pass through the origin. Besides, the lobe areas of the pinched hysteresis loops shrink as the frequency increases [31], thereby their fingerprints are confirmed.

### 2.1. Parametric Representation and DC V−I Curve

According to the description on the obove natural characteristics, the number and location of locally active domains are determined by the parameter *A*. Herein, the there cases (i.e., $A=A_{M}$, $A=A_{FS}<A_{M}$, and $A=A_{FL}>A_{M}$) are discussed and their DC V−I curves be drawn as follows:

Case 1: $A=A_{M}$

when N=1, m=2, a=0, $n_{1}=6$, $h_{1}=12$, $A=A_{M}=9$, the simplest 2− lobe CCM can be obtained and rewritten as:

(2) $$\left\{\begin{array}{c} i=G_{0}x^{2}\cdot v\\ \frac{dx}{dt}=9-x+\left|x-6\right|-\left|x-12\right|+v. \end{array}\right.$$

**Figure 2:**
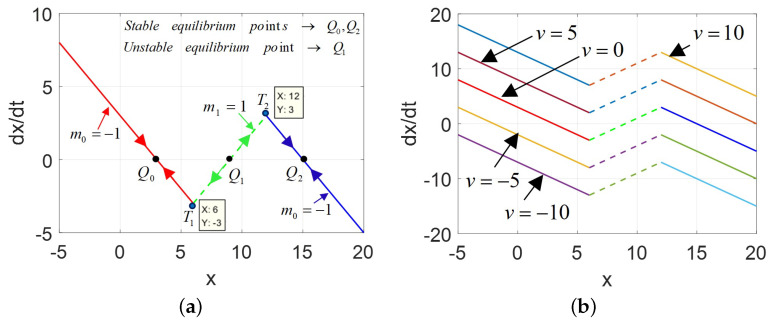
DRM of the proposed model, $A=A_{M}$: (**a**) the DRM for v= 0 V (power-off plot), (**b**) the DRM for v= 0 V, ±5 V, ±10 V.

Then, the dynamic route maps (DRM) of the model (2) are shown in Figure 2.

From Figure 2a, when 2-lobe CCM is short-circuited (v=0), the direction of motion of the state variable *x* from any initial state x(0) is indicated by the arrowheads on the power-off-plot (POP). The features of two turning points (namely, $T_{1},T_{2}$) and three equilibrium points (i.e., $Q_{0}\left(x=3\right),Q_{1}\left(x=9\right),Q_{2}\left(x=15\right)$) are identical with the description in Proposition 2 (1)∼(3). Both $Q_{0}$ and $Q_{2}$ are stable equilibrium points, whereas $Q_{1}$ is unstable due to the state variable x(t) diverges away from $Q_{1}$. The dynamic routes for v=0,±5,±10 are depicted in Figure 2b. Each DRM route is divided into 3 segments by breakpoints at x=6 and x=12, which has two asymptotically stable and one unstable equilibrium points. Moreover, two enclosed areas can be observed from Figure 2a, i.e., areas $\Delta_{011}$ and $\Delta_{122}$ formed by points $Q_{0}$, $T_{1}$, and $Q_{1}$ and $Q_{1}$, $T_{2}$, $Q_{2}$ respectively.

Generally, the DC V−I curve is used to make sure the basic parameters for the proposed CCM. Therefore, the explicit formula is obtained from Equation (2), which equals zero and computes $G_{0}=0.1$, x=X as the functions of v=V and i=I,

(3) $$\left\{\begin{array}{c} I=G_{0}X^{2}\cdot V\\ f_{2}\left(X,V\right)=9-X+\left|X-6\right|-\left|X-12\right|+V. \end{array}\right.$$

**Figure 3:**
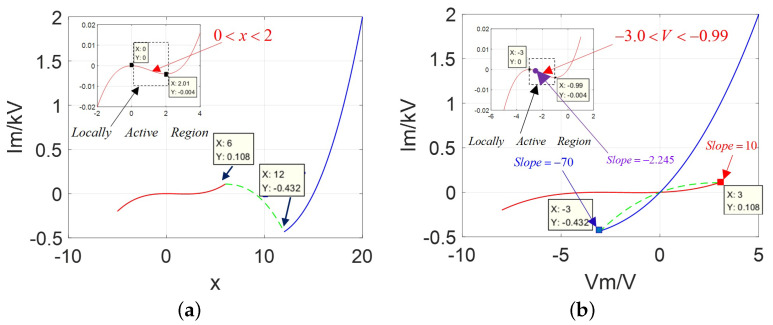
Graphs of current against state variable and DC V−I curve: (**a**) Ivs.X with only one negative slope domain in the first stable branch over the variable range 0<x<2; (**b**) Ivs.V with only one negative slope domain over the variable range −3 V <v< −0.99 V.

The corresponding DC V−I curves are drawn in Figure 3 over the input variable range x∈[−10,20] and voltage range V∈[−10,5].

In Figure 3, abserved from between Figure 3a,b, the DC V−I curve emerges only one negative slope domain in the first stable branch over the positive variable ranges 0<x<2 and the negative voltage range −3 V <v< −1.0 V, which lies in the third quadrant of the $V_{m}-I_{m}$ coordinates and implies the existence of the locally active region. It satisfies the description in case (1). Besides, in Figure 3b, the slope at $V_{p}=$−2.5 V is −2.245 (calculated from the coordinates at $V_{p}=$−2.49 V and $V_{p}=$−2.51 V) in purple; the slope $V_{b}=$−2.99 V is −70 (given from the coordinates at $V_{b}=$−2.98 V and $V_{b}=$−3.0 V) in blue; the slope $V_{r}=$ 2.9 V is 10 (got from the coordinates at $V_{r}=$ 2.91 V and $V_{r}=$ 2.89 V) in red.

Observed from Equation (1) and Figure 3, when the parameters A=30, $n_{j}=20$, $h_{j}=40$ are chosen, the proposed universal model is equivalent to the classical CCM model, which satisfies the rule $A=A_{M}$ and conforms to the description on its natural features. That means their DC V−I curves have two stable branches and one unstable branch, and only one negative slope domain in the first branch over the negative voltage range; both upper left lobe and lower right lobe have similar shapes and areas (called a symmetrical “Corsage ribbon”). Hence, they could be considered as belonging to the same family of universal 2-lobe CCM.

Case 2: $A=A_{FS}<A_{M}$

In this case, we choose a new group of parameters ($n_{j}=6$, $h_{j}=22$, m=2, $A=A_{FS}<A_{M}=9$), the proposed model is restated as follows:

(4) $$\left\{\begin{array}{c} i=G\left(x\right)v=G_{0}x^{2}\cdot v\\ \frac{dx}{dt}=9-x+\left|x-6\right|-\left|x-22\right|+v. \end{array}\right.$$

**Figure 4:**
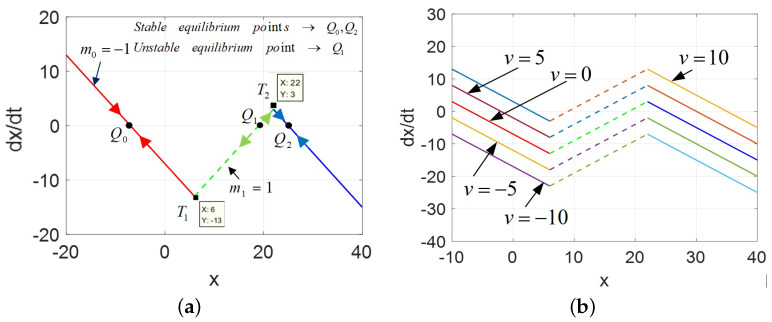
The DRM of 2-lobe CCM, $A=A_{FS}<A_{M}$: (**a**) DRM for the POP, v=0; (**b**) DRM for v=0,5,10.

The DRM for the POP and v=0,5,10 are plotted in Figure 4. Comparing Figure 4a with Figure 2a, the area $\Delta_{011}$ enclosed by three points (i.e., $Q_{0},T_{1},and\;Q_{1}$) is much larger than $\Delta_{122}$ by three points (i.e., $Q_{1},T_{2},and\;Q_{2}$), which implies the different shapes and areas of the corsage ribbon.

**Figure 5:**
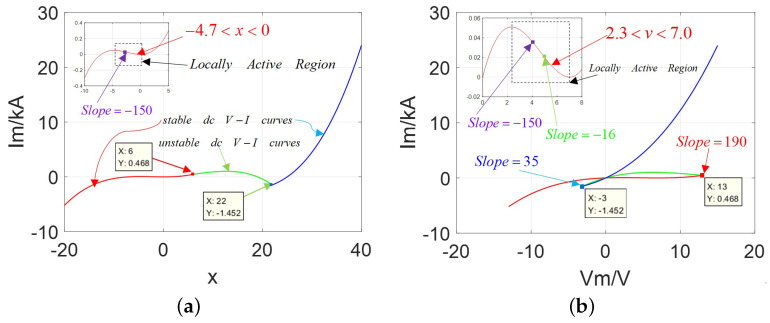
Graphs of current against state variable and DC V−I curve: (**a**) Ivs.X with only one negative slope domain in the over the range −4.7<x<0; (**b**) Ivs.V with the negative slope domain over the variable range 2.3 V <v< 7.0 V.

With the new parameters, the graphs of Ivs.X and DC V−I are redrawn and shown in Figure 5. There is only one negative slope domain in the first branch over the positive voltage ranges 2.3 V <v< 7.0 V in Figure 5b, which lies in the first quadrant of the $V_{m}-I_{m}$ coordinates and implies the existence of locally active regions. It satisfies the description in case (2) of Proposition 1. Then, several certain slopes are calculated, such as $V_{p}=$ 4.0 V is −150 in the purple point, $V_{g}=$ 5.0 V is −16 in the green point, $V_{b}=$ −3.0 V is 35 in the blue point, and $V_{r}=$ 13.0 V is 190 in the red point.

Observed from Figure 5, the upper left lobe becomes much bigger than the lower right one, which is called the asymmetrical “Corsage ribbon”.

Case 3: $A=A_{FL}>A_{M}$.

The third group of parameters ($n_{j}=6$, $h_{j}=22$, m=2, and $A=A_{FL}=20>A_{M}$) are given for showing the more active and locally active domains clearly, the proposed model is recast as follows:

(5) $$\left\{\begin{array}{c} i=G\left(x\right)v=G_{0}x^{2}\cdot v\\ \frac{dx}{dt}=20-x+\left|x-6\right|-\left|x-22\right|+v. \end{array}\right.$$

**Figure 6:**
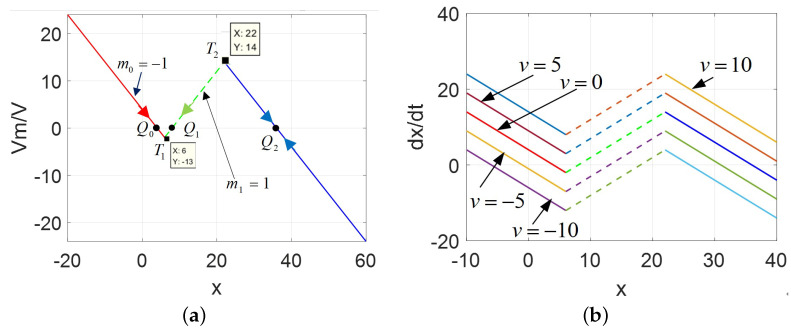
The DRM of 2-lobe CCM, $A=A_{FL}>A_{M}$: (**a**) DRM for the POP; (**b**) DRM for v=0,5,10.

The DRM for the POP and v=0,5,10 are plotted in Figure 6.

**Figure 7:**
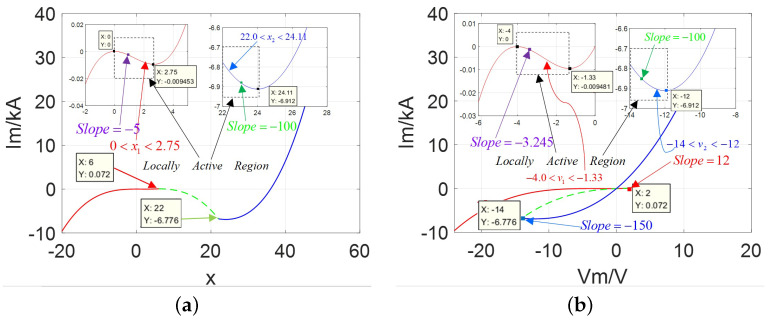
Loci of Ivs.X and DC V−I curves: (**a**) Ivs.X with only one negative slope domain in the over the variable range 0<x<2.75 and 22<x<24.11; (**b**) Ivs.V with the negative slope domain over the variable range −4.0 V <v< −1.33 V and −14.0 V <v<−12.0 V.

Comparing Figure 6a with Figure 2a, the area $\Delta_{011}$ enclosed is much smaller than the area $\Delta_{122}$, which is also different from the previous cases. It is not only multi-valued, very unusually, but resembles an asymmetrical phenomenon. Moreover, the graphs of the Ivs.X and DC V−I curves are depicted in Figure 7.

It can be seen from Figure 7, there are two negative slope domains in the stable branches over the ranges of −4.0 V <v< −1.33 V and −14.0 <v< −12.0 V via the red and blue solid lines, which indicate the existence of two locally active regions and both lie in the third quadrant of the $V_{m}-I_{m}$ coordinates. This situation satisfies the description of the case 3 in the above section. Additionally, several important slopes can be calculated at $V_{p}=$ −3.5 V is −3.245 in the purple point, $V_{g}=$ −13.6 V is −100 in the green point, $V_{b}=$ −14.0 V is −150 in the blue point, and $V_{r}=$ 2.0 V is 12 Siemens in the red point. Therefore, the asymmetrical “Corsage ribbon” can emerge.

**Figure 8:**
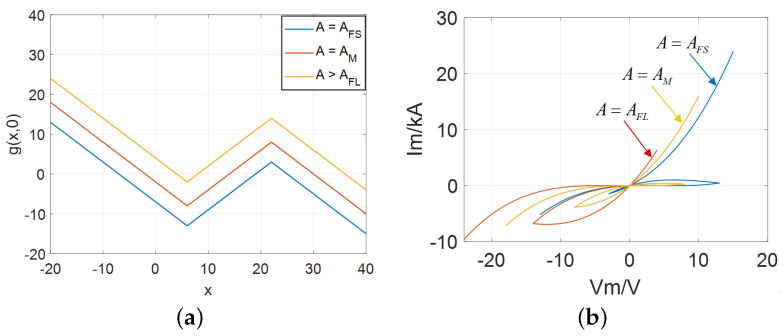
The DRM and phase locus with $A_{FS}$, $A_{M}$, and $A_{FL}$: (**a**) DRMs along v=0; (**b**) phase trajectory in Ivs.V.

To summarize, the curves of DRMs and phase portraits with different cases (i.e., $A_{FS}$, $A_{M}$, and $A_{FL}$) are illustrated in Figure 8.

Based on the previous description, the following conclusion can be drawn. When the other parameters (such as *m*, $n_{j}$, and $h_{j}$) are fixed: (1) the shape and symmetry of the lobes, stability, locally active domains depend on the value of parameter *A*; (2) in order to obtain the negative slope domains, the ranges of the variables *x* and *v* at the equilibrium point (*Q*) obey the condition: xv<0.

### 2.2. The Generation of Multiply Lobes for the Universal CCMs

The proposed *m*-lobe CCM can be implemented by model (1). The number of lobes can be captured and their parameters are listed in Table 1.

**Figure 9:**
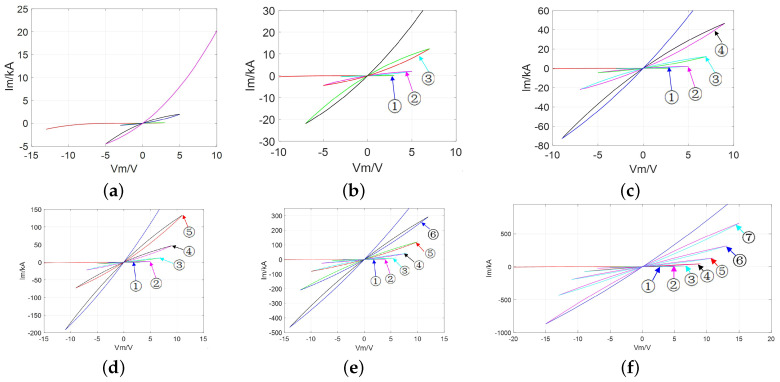
Phase trajectory in Ivs.V: (**a**) m=4; (**b**) m=6; (**c**) m=8; (**d**) m=10; (**e**) m=12; (**f**) m=14.

Additionally, the generation of *m*-lobe CCMs are shown in Figure 9. According to the above description, it can be seen that the local active domain (LAD) in the first stable branch lobes might be impacted but will not disappear with the increase of the number of lobes. Moreover, for any *m*-lobe CCM, the feature of parameter *A* will not change as the number of lobes increases, which is in line with statements on the natural characteristics of the CCM.

## 3. Small-Signal Equivalent Model and Edge of Chaos

### 3.1. Small-Signal Equivalent Model with One Capacitor

In order to predict the response of a memristor to a small-signal input applied at an equilibrium point, the small-signal equivalent of the CCM has been designed by L.O Chua and his coworkers in 2015. They have also pointed out that adding at least one energy storage element across the Chua Corsage Memristor could make sure the circuit oscillates. In these papers, the analysis on an inductance and two resistances were elaborated. Moreover, they have verified that a positive inductance is needed to compensate for the imaginary part of admittance Y(iω,V) as well as to make the total impedance to zero. As standard electronic circuit theory, some conclusion can be directly obtained via impedance function instead of admittance, which is our focus in this subsection.

The small-signal impedance (Z(s,V)) at the equilibrium points can be summarized and uncovered as follows:

(6) $$Z\left(s,V\right)=\frac{1}{a_{12}\left(V\right)}\cdot \frac{s-b_{11}\left(V\right)}{s-b_{11}\left(V\right)+\frac{a_{11}\left(V\right)}{a_{12}\left(V\right)}b_{12}\left(V\right)},$$

where $a_{11}$, $a_{12}$, $b_{11}$, $b_{12}$ express the parameters of the Laplace transformation for $\delta i_{m}\left(t\right)$ and $\delta v_{m}\left(t\right)$, which neglects the higher-order terms by $\left|\delta i\right|\ll 1$ and $\left|\delta v\right|\ll 1$. Then,

(7) $$\left\{\begin{array}{c} \begin{array}{cc} a_{11}\left(V\right)=v\frac{\partial G\left(x\right)}{\partial x}\left|{}_{Q}\right.=2XV, & a_{12}\left(V\right)=G\left(x\right)\frac{\partial v}{\partial v}\left|{}_{Q}\right.=X^{2} \end{array}\\ b_{12}\left(V\right)=\frac{\partial g\left(x,v\right)}{\partial v}\left|{}_{Q}\right.=1\\ b_{11}\left(V\right)=\frac{\partial g\left(x,v\right)}{\partial x}\left|{}_{Q}\right.=\left\{\begin{array}{cc} -1 & \begin{array}{cccc} if & X<n_{j} & or & X>h_{j} \end{array}\\ 1 & \begin{array}{ccc} if & n_{j}\leq X\leq h_{j} & unstable. \end{array} \end{array}\right. \end{array}\right.$$

The following equivalent circuit form is rewritten from Equation (6):

(8) Zs,V=s+−b11Vs+−b11V+a11Vb12Va12Q·1a12Vκ=11X2X2,p=−1+2V2VXX,z=−1.

Then, the small-signal equivalent circuit with one capacitor and two equivalent resistors can be designed,

(9) Zs,V=s+11RmCmRmCms+11Rm+RnCmRm+RnCm·RnRmRm+RnCm=−2XV,Rm=−112XV2XVRn=11X2+2XVX2+2XV.

**Figure 10:**
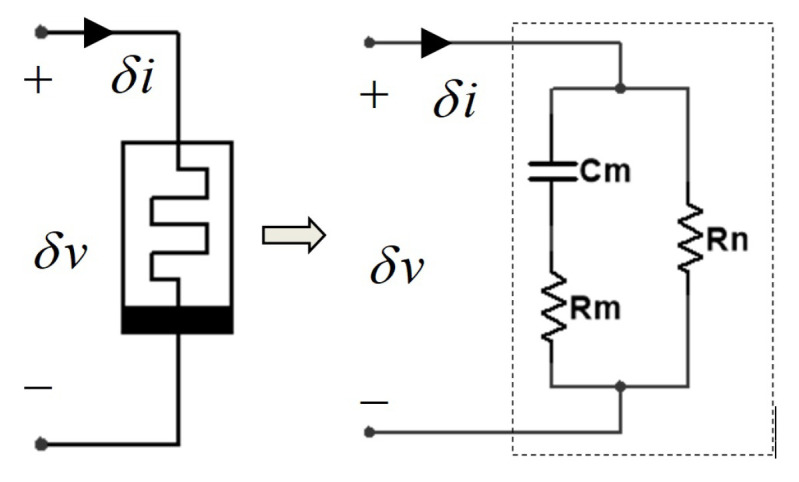
The schematic diagram of one novel small-signal equivalent circuit.

**Figure 11:**
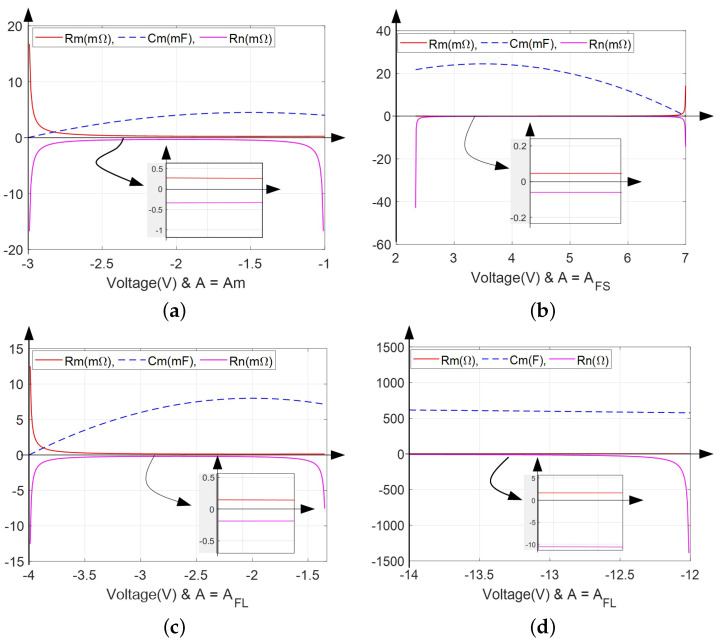
Plot of the small-signal inductance $C_{m}$ and resistances $R_{m}$ and $R_{n}$ over the negative slope domains: (**a**) $A=A_{M}$; (**b**) $A=A_{FS}$; (**c**) $A=A_{FL}$ in LAD1; (**d**) $A=A_{FL}$ in LAD2.

Besides, the schematic diagram of the small-signal equivalent circuit of the impedance function with a capacitor ($C_{m}$) and two resistors ($R_{m}$ and $R_{n}$) is drawn in Figure 10. Then, the curves defining the parameters of this novel small-signal circuit model are shown in Figure 11. Both $C_{m}$ and $R_{m}$ are positive and $R_{n}$ is negative.

Since the small-signal impedance Z(s,V) at v=V has only a real zero $s=b_{11}\left(V\right)$, which is necessary to add at least one capacitor (or inductor) across the Chua Corsage Memristor in order to make the circuit oscillate at some frequency $\omega_{0}>0$. Its universal impedance function can be expressed as follows

(10) $$Z\left(s,V\right)=\kappa \frac{s-z}{s-p}=\frac{R_{n}R_{m}}{R_{m}+R_{n}}\cdot \frac{s+\frac{1}{R_{m}C_{m}}}{s+\frac{1}{\left(R_{m}+R_{n}\right)C_{m}}}$$

where, $k=R_{m}R_{n}/\left(R_{m}+R_{n}\right)$ is represented as the gain of impedance, $p=-1/[\left(R_{m}+R_{n}\right)C_{m}]$ is denoted the only real pole, and z=−1 is only real zero for the proposed universal *m*-lobe CCM.

To determine the type of energy-storage element (inductor or capacitor), the following frequency response Z(iω,V) should be derived and plotted by substituting s=iω. The frequency response of impedance function, Re[Z(iω,V)] and Im[Z(iω,V)] are given in Equation (11):

(11) ReZiω,V=RnRm+RnRmCm2ω2+1Rm+RnCmω2+1ImZiω,V=ω2Rm+RnRnCmRm+RnCmω2+1.

Observed from Figure 11, we can clearly see that the configured small-signal equivalent circuit and the analysis on its impedance function verify the oscillation theory and the existence of oscillators from another perspective.

### 3.2. Edge of Chaos

Based on the representation of zeros and poles in the impedance function, the proposed model clearly exhibit the edge of chaos as shown on the zero-pole diagrams. Observed from Figure 3, Figure 5 and Figure 7, different negative slopes regions can be captured by adjusting parameter *A*. The relationship of parameters *x* and *v* conforms to the rule *x*v<0.

Besides, the feature of parameter *A* can be summarized as: when $A\leq A_{M}$, there exists only one negative slope region; when $A>A_{M}$, there are two.

**Figure 12:**
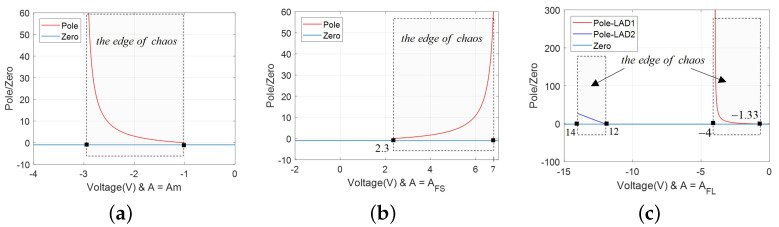
The zeros *z* and pole *p* of the model (1): (**a**) $A=A_{M}$, v∈(−3,−0.99) in LAD; (**b**) $A=A_{FS}$, v∈(2.3,7.0) in LAD; (**c**) $A=A_{FL}$, $v_{1}\in \left(-4.0,-1.33\right)$ in LAD1 and $v_{2}\in \left(-14.0,-12.0\right)$ in LAD2.

Next, the gain, zero and pole of the Equation (9) are calculated and the diagrams are plotted in Figure 12 to exhibit the edge of chaos.

(12) κ=X−2,p=−1+2V2VXX,z=−1.

From Figure 12, the distribution of LADs are identical with the DC V−I curves in Figure 3, Figure 5 and Figure 7. There is also one energy storage element that is indeed required for generating an oscillator, which could be an inductor or capacitor in serial/parallel with the resistor.

Based on the edge of chaos and Figure 11 and Figure 12, Re[Y(iω,V)]<0 could occur for ω∈(−∞,+∞). That is, the conditions can be satisfied with components $C_{m}>0$, $R_{m}>0$, $R_{n}<0$, $|R_{n}|>R_{m}$.

## 4. The Simplest Oscillator and Its Neural Oscillation

In light of the concepts and techniques of both electric circuit and nonlinear dynamics theory, there is a pair of complex-conjugate poles on the imaginary axis at some frequency ω>0 in one oscillator circuit. According to [1,2,3,11,14], one external inductance ($L^{*}$) can be chosen to present plenty of nerual oscillation. In this section, the discussion on the properties and rules for this positive inductance are given as well as its frequency response and neural dynamics behaviors.

**Figure 13:**
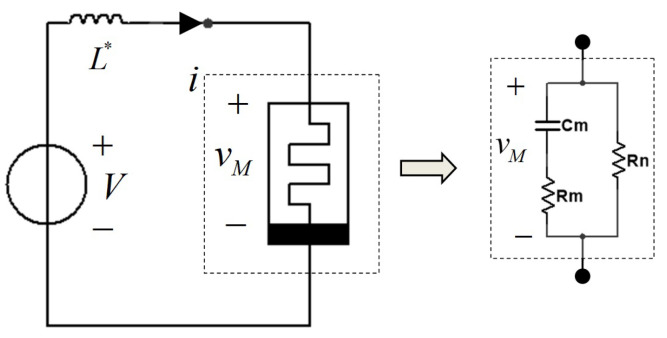
Oscillator circuit of universal *m*-lobe CCM oscillator.

In Figure 13, $L^{*}$ is an external positive inductance, $v_{M}$ is considered the voltage of the proposed universal *m*-lobe CCM; *V* represents a DC voltage source.

### 4.1. The External Positive Inductance

Based on the frequency response of the derived impedance function (Z(iω,V)) and the relationship between admittance and impedance (Y(iω,V)=1/Z(iω,V)), the external inductances ($L^{*}$) can be calculated in three cases.

Case 1: $A=A_{M}$

**Figure 14:**
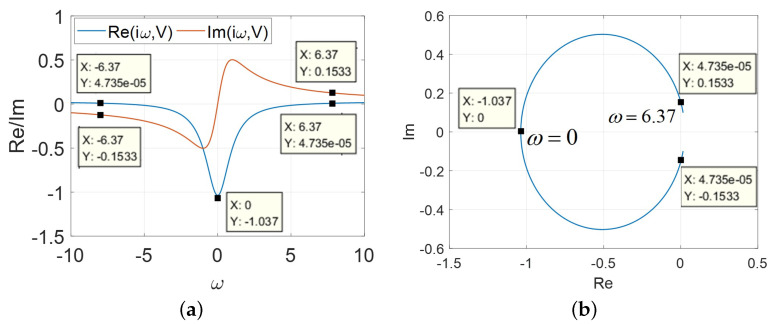
The frequency response, $A=A_{M}$: (**a**) Re[Y(iω,V)] and Im[Y(iω,V)] over the ranges $-10<\omega^{*}<10$; (**b**) $Re[Y\left(i\omega^{*},V\right)]$vs.$Im[Y\left(i\omega^{*},V\right)]$.

The frequency response for the model (2) are illustrated in Figure 14. The admittance *Y* are shown in Figure 14a over the frequency range −50 $\mathrm{rad}/s\leq \omega^{*}\leq$ 50 rad/s.

From Figure 14a, the $Re[Y\left(i\omega^{*}\right)]=0$ at $\omega^{*}=\pm 6.37$ rad/s whereas $Im[Y\left(i\omega^{*}\right)]=\pm 0.1533$ with the inductance of $L^{*}$ are calculated via Chua’s formula as follows:

(13) L*=1ω*ImYω*=16.37×0.1533=1.024H.

From Figure 14b, it is noted that $L^{*}$ could compensate the imaginary part of impedance when the voltage lies in the LAD. Then, several important points are computed as: $Im[Y\left(i\omega^{*}\right)]=0$, $Re[Y\left(i\omega^{*}\right)]=-1.037$ at $\omega^{*}$ =0 rad/s; $Im[Y\left(i\omega^{*}\right)]=\pm 0.1533$, $Re[Y\left(i\omega^{*}\right)]$=0. at $\omega^{*}=\pm 6.37$ rad/s, respectively. When these points lie in the open right-half plane (RHP), and the destabilization of the oscillator circuit could be demonstrated by the inductor ($L^{*}$).

Case 2: $A=A_{FS}<A_{M}$

**Figure 15:**
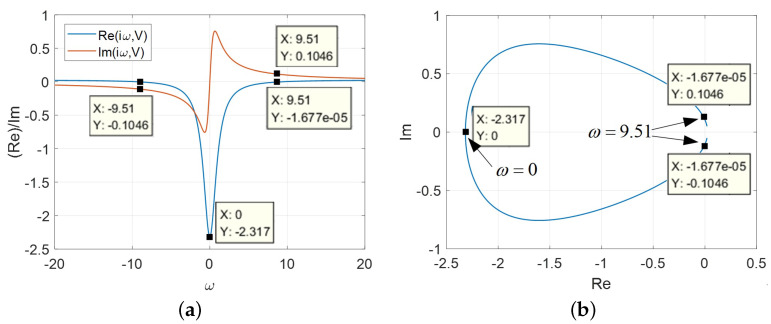
The frequency response ($A=A_{FS}<A_{M}$): (**a**) Re[Y(iω,V)] and Im[Y(iω,V)] for $-20<\omega^{*}<20$; (**b**) $Re[Y\left(i\omega^{*},V\right)]$vs.$Im[Y\left(i\omega^{*},V\right)]$.

In this case, the frequency response of the model is illustrated in Figure 15. The complex admittance $Y(i\omega^{*},V)$ are plotted in Figure 15a over the frequency range of −50 rad/s $\leq \omega^{*}\leq$ 50 rad/s. The $Re[Y\left(i\omega^{*}\right)]=0$ at $\omega^{*}=\pm 9.51$ rad/s whereas $Im[Y\left(i\omega^{*}\right)]=\pm 0.1046$ with the external $L^{*}$ are calculated as follows:

(14) L*=1ω*ImYω*=19.51×0.1046=1.005H.

Observed from Figure 15b, when $L^{*}=1.005H$ is chosen and the voltage lies in the LAD, the imaginary part can be compensated. Some parts of the loci shown in Figure 15b are located in the open RHP, which implies that the circuit can be destabilized by varying the inductance $L^{*}$ to generate the desired nonlinear behaviors.

Case 3: $A=A_{FL}>A_{M}$

**Figure 16:**
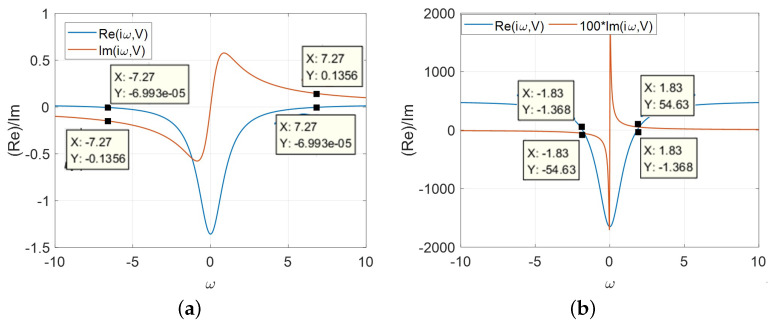
The frequency response, $A=A_{FL}>A_{M}$, Re[Y(iω,V)] and Im[Y(iω,V)]: (**a**) v∈ LAD1; (**b**) v∈ LAD2.

In this case, the frequency response curves obtained from both LAD1 and LAD2 are shown in Figure 16. For *v* in LAD1, the oscillation occurs when $\omega^{*}=\pm 7.27$ rad/s with $Y_{1}=0\pm 0.1356i$ and $\omega^{*}=\pm 1.83$ rad/s with $Y_{2}=0\pm 0.5463i$, respectively. The corresponding inductance $L^{*}$ for both LADs can be calculated by the following formulas:

(15) L1*=1ω*Im1Yω*=17.27×0.1356=1.0144HL2*=1ω*Im2Yω*=11.83×54.63=10.0027mH.

According to Equations (13)–(15), the following conclusion can be reached: in the simplest oscillator circuit, in order to compensate for the imaginary part of admittance and capture the nonlinear behaviors, the external positive inductance ($L^{*}$) always need to be connected in series with all types of the small-signal equivalent circuits. The number and distribution of $L^{*}$ is determined by the parameter *A* and the LAD(s).

Subsequently, it is summed up as follows: when the parameter $A\leq A_{M}$, only one external inductor ($L^{*}$) is needed to compensate the imaginary part of admittance and generate the neural oscillation. Whereas, when the parameter $A>A_{M}$, more than one inductance value can be used to design the oscillator.

### 4.2. Oscillation Mechanism

The state equations of the simplest oscillator circuit in Figure 13 can be written as follows:

(16) $$\left\{\begin{array}{c} \frac{dx}{dt}=A-x+\sum_{j=1}^{N}\left(\left|x-n_{j}\right|-\left|x-h_{j}\right|\right)+\frac{i}{G_{0}x^{2}}\\ \frac{di}{dt}=\frac{1}{L^{*}}\left(V-\frac{i}{G_{0}x^{2}}\right), \end{array}\right.$$

where *A*, $n_{j}$, and $h_{j}$ denote the parameters; *x* and *i* are the state variable and the current of the inductor ($L^{*}$); $L^{*}$ is a positive inductance, and $G_{0}=0.1$. Then, the output voltage ($v_{out}$) is set as the voltage of the universal *m*-lobe CCM, that is, $v_{out}=-v_{M}$.

To ease the demonstration of local activity and edge of chaos, from which we can identify the mechanism of the action potential in this circuit, the following universal equation of $Y_{2}\left(s,V\right)$ at the equilibrium point *Q* for the oscillator circuit is formulated:

(17) $$Y_{2}\left(s,V\right)=\frac{1}{Z_{2}\left(s,V\right)}=\frac{1}{L^{*}}\cdot \frac{s+\frac{1}{\left(R_{m}+R_{n}\right)C_{m}}}{s^{2}+\frac{R_{n}R_{m}C_{m}+L^{*}}{\left(R_{m}+R_{n}\right)C_{m}L^{*}}s+\frac{R_{n}}{\left(R_{m}+R_{n}\right)C_{m}L^{*}}}=\kappa_{2}\frac{s-z_{2}}{\left(s+p_{21}\right)\left(s-p_{22}\right)},$$

where the impedance of an external positive inductance ($L^{*}$) is represented as $Z_{L^{*}}=L*s$. The zeros and pole of $Y_{2}\left(s,V\right)$ are solved as follows:

(18) p21,22=z22L*RnRmCm+L*∓RnRmCm+L*2+4RnL*4RnL*z2z2κ2=11L*L*>0,z2=−11Rm+RnCmRm+RnCm.

From Equation (18), both poles $p_{21}$ and $p_{22}$ are the functions of the external positive inductance ($L^{*}$). When the condition ${\left(R_{n}R_{m}C_{m}+L^{*}\right)}^{2}+4R_{n}L^{*}/z_{2}<0$ is satisfied, an oscillator emerges. Correspondingly, the frequency response can be moved from the open LHP to the open RHP by increasing the inductance $L^{*}\in \left[0,+\infty \right)$.

The frequency response $Y_{2}\left(i\omega ,V\right)$ is derived by substituting s=iω in Equation (19):

(19) Y2iω,V=ReY2iω,V+iImY2iω,VReY2iω,V=−z2·ω2RmCm−z2RnL*L*ω2+z2Rn2+z2RnRmCm+z2L*2ω2ImY2iω,V=−ωL*L*ω2+Rnz2+z22RnRmCm+L*L*ω2+z2Rn2+z2RnRmCm+z2L*2ω2.

*Notably* for the second-order oscillator circuit with the CCM, the loci of $Re[Y_{2}\left(s,V\right)]$, $Im[Y_{2}\left(s,V\right)]$ and frequency response of $Y_{2}\left(i\omega ,V\right)$ have been analyzed [1,2,10,11,12,13,28], which could exhibit the commonality of the universal *m*-lobe CCM. Therefore, they are omitted in this paper.

According to the admittance function and frequency response of Figure 13, Hopf bifurcation, stable, and destabilized phenomena might exist, as well as the spiking and neural dynamics, which are the main focus in the next subsection.

### 4.3. Oscillation and Neural Dynamics

(1) Limit cycle and supercritical Hopf bifurcation.

**Figure 17:**
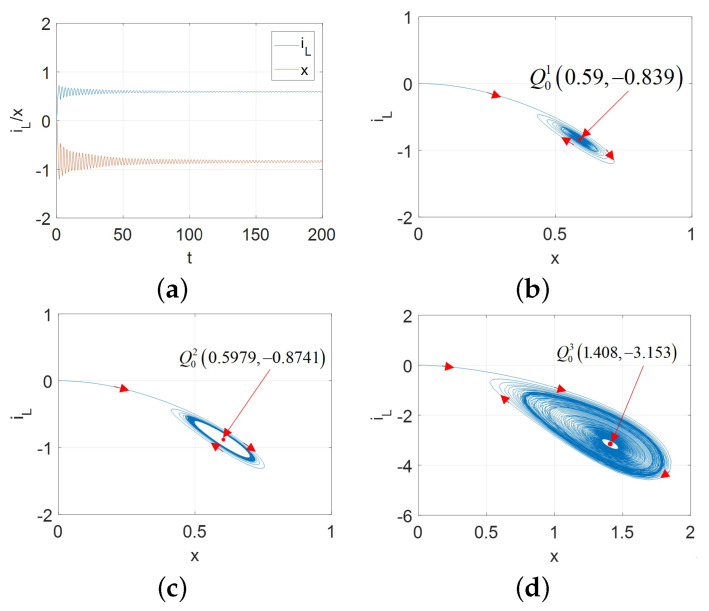
The supercritical Hopf bifurcation over the range vϵ[−2.4,−1.6] and initial condition $[x\left(0\right),i_{L}\left(0\right)]=\left[0.01,0\right]$ for $A=A_{M}$: (**a**) x(t) and $i_{L}\left(t\right)$ for v=−2.41 V; (**b**) stable limit cycle converges to $Q_{0}^{1}$ (0.59, –0.839) for v=−2.41; (**c**) stable limit cycle corresponding to the periodic waveforms for v=−2.39; (**d**) stable limit cycle converges to $Q_{0}^{3}$ (1.408, –3.153) for v=−1.5906.

**Figure 18:**
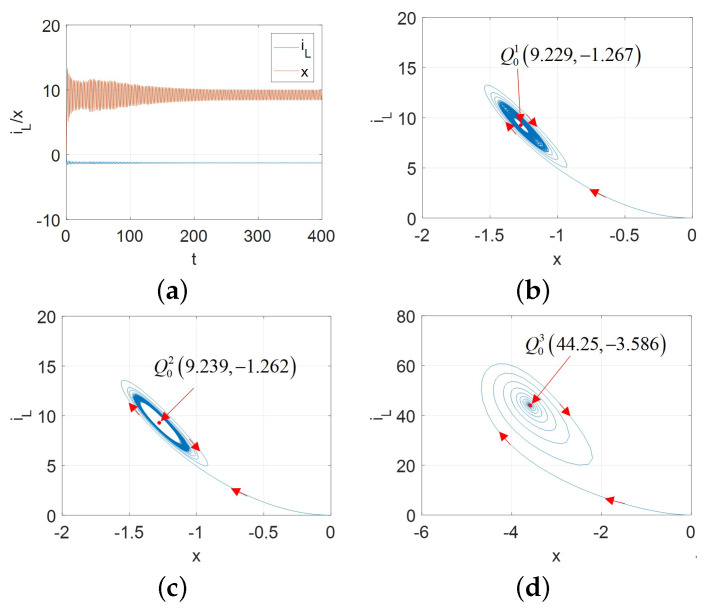
vϵ[3.6,5.725] and initial condition $[x\left(0\right),i_{L}\left(0\right)]=\left[-0.01,0\right]$ for $A=A_{FS}$: (**a**) x(t) and $i_{L}\left(t\right)$ for v= 5.73 V; (**b**) stable limit cycle converges to $Q_{0}^{1}$ (9.229, –1.267) for v=5.73; (**c**) stable limit cycle corresponding to the periodic waveforms for v=5.72; (**d**) stable limit cycle converges to $Q_{0}^{3}$ (44.25, –3.586) for v=3.4.

**Figure 19:**
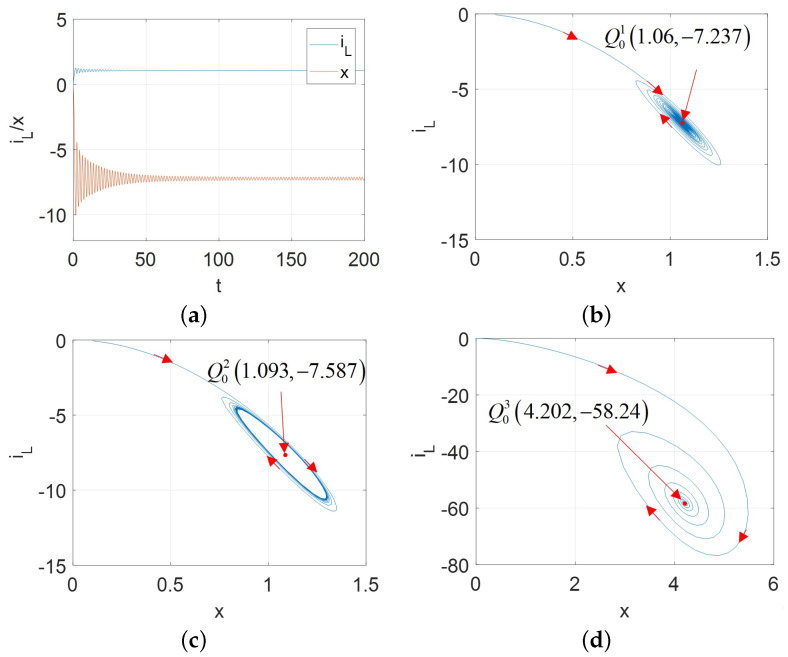
$v_{1}\epsilon \left[-2.155,-1.5\right]$ and initial condition $[x\left(0\right),i_{L}\left(0\right)]=\left[0.1,0\right]$ for $A=A_{FL}$: (**a**) x(t) and $i_{L}\left(t\right)$ for v=−2.17 V; (**b**) stable limit cycle converges to $Q_{0}^{1}$ (1.06, −7.237) for $v_{1}=-$2.17 V; (**c**) stable limit cycle corresponding to the periodic waveforms for $v_{1}=-$2.0 V; (**d**) stable limit cycle converges to $Q_{0}^{3}$ (4.202, −58.24) for v=−1.4.

**Figure 20:**
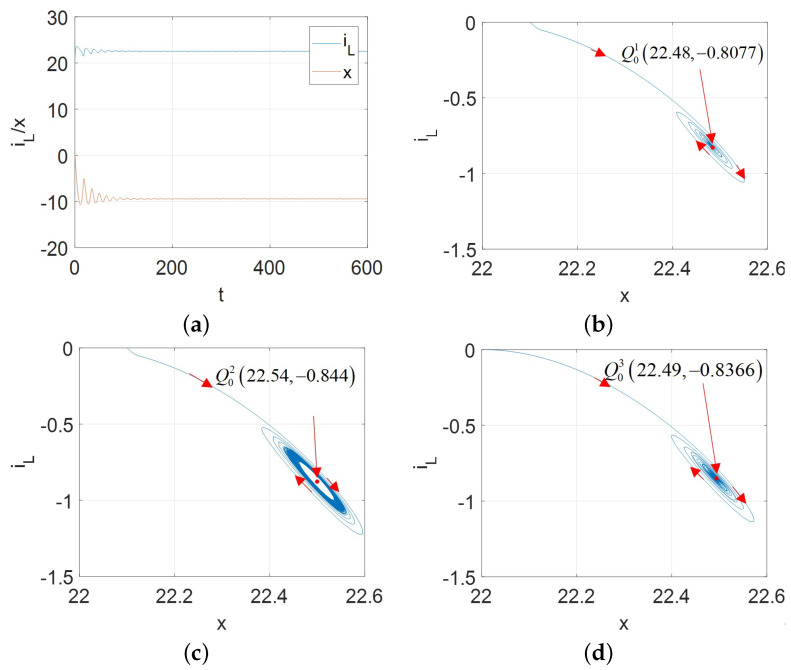
$v_{2}\epsilon \left[-13.42,-13.05\right]$ and initial condition $[x\left(0\right),i_{L}\left(0\right)]=\left[10,0\right]$ for $A=A_{FL}$: (**a**) x(t) and $i_{L}\left(t\right)$ for v=−13.5 V; (**b**) stable limit cycle converges to $Q_{0}^{1}$ (22.48, −0.8077) for $v_{1}=-$13.5 V; (**c**) stable limit cycle corresponding to the periodic waveforms for $v_{1}=-$13.3 V; (**d**) stable limit cycle converges to $Q_{0}^{3}$ (22.49, –0.8366) for v=−12.8.

Hopf bifurcation gives birth to a limit cycle to change the nonlinear system stability [2,13]. A stable limit cycle could lead to the supercritical Hopf bifurcation. Therefore, for the proposed universal CCM, its oscillators can exhibit the supercritical Hopf bifurcation as shown in Figure 17 for $A=A_{M}$, Figure 18 for $A=A_{FS}$, Figure 19 and Figure 20 for $A=A_{FL}$.

Observed from Figure 17, Figure 18, Figure 19 and Figure 20, the limit cycle lies in the open RHP of its pole plot between two Hopf bifurcation points. Therefore, it can be confirmed that the stable limit cycle does appear.

(2) Neural oscillation

**Figure 21:**
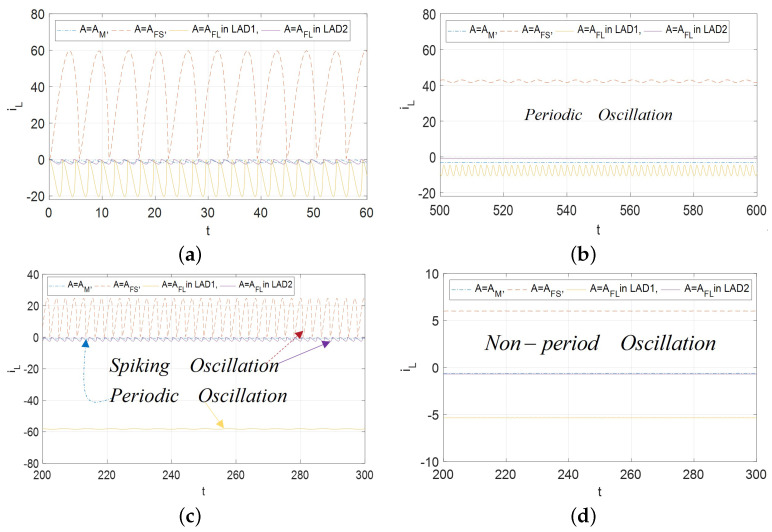
The oscillation behaviors: (**a**) Non-sinusoidal periodic waveforms at v=−1.65 V for $A=A_{M}$ in LAD is drawn by blue dotted solid line, at v=5.62V for $A=A_{FS}$ in LAD is drawn by red dotted line, at v=−1.62 V for $A=A_{FL}$ in LAD1 is drawn by yellow solid line, at v=−13.1 V for $A=A_{FL}$ in LAD2 is drawn by purple solid line; (**b**) Periodic oscillation waveforms v=−1.48 V for $A=A_{M}$ in LAD is drawn by blue dotted solid line, v= 3.55 V for $A=A_{FS}$ in LAD is drawn by red dotted line, v=−1.62 V for $A=A_{FL}$ in LAD1 is drawn by yellow solid line, v=−13.045 V for $A=A_{FL}$ in LAD2 is drawn by purple solid line; (**c**) Multiplex-oscillator waveforms v=−2.39 V for $A=A_{M}$ in LAD is drawn by blue dotted solid line, v= 5.4 V for $A=A_{FS}$ in LAD is drawn by red dotted line, v=−2.145 V for $A=A_{FL}$ in LAD1 is drawn by yellow solid line, v=−13.4 V for $A=A_{FL}$ in LAD2 is drawn by purple solid line; (**d**) Non-periodic oscillation waveforms v=−2.5 V for $A=A_{M}$ in LAD is drawn by blue dotted solid line, v= 6.0 V for $A=A_{FS}$ in LAD is drawn by red dotted line, v=−2.2 V for $A=A_{FL}$ in LAD1 is drawn by yellow solid line, v=−12.3 V for $A=A_{FL}$ in LAD2 is drawn by purple solid line.

When the memristive emulator is utilized to mimic one biological neuron, spiking oscillation can be considered one of the most prominent phenomena. During the supercritical Hopf bifurcation intervals, the large-amplitude non-sinusoidal periodic waveform can be discovered in three cases, which is shown in Figure 21.

From Figure 21a, the non-sinusoidal periodic oscillation occurs in an open region v∈[−2.35,−1.60] for $A=A_{M}$ as depicted by the blue solid line (or v∈[5.25,5.669] for $A=A_{FS}$ as depicted by red dotted line or $v_{1}\in \left[-2.151,-2.352\right]$ as depicted by yellow solid line and $v_{2}\in \left[-13.385,-13.286\right]$ as depicted by purple solid line for $A=A_{FL}$) on the RHP. Then, the periodic oscillation exists in an open region v∈[−1.45,−1.60) for $A=A_{M}$ as depicted by blue solid line (or v∈[3.22,3.25) for $A=A_{FS}$ as depicted by red dotted line or $v_{1}\in \left(-1.33,-2.03\right)$ as depicted by yellow solid line and $v_{2}\in \left(-13.049,-13.042\right)$ as depicted by purple solid line for $A=A_{FL}$) on the LHP in Figure 21b. However, in Figure 21c, the spiking oscillation is observed in $A=A_{FS}$ in LAD and $A=A_{FL}$ in LAD2 domains whereas the periodic oscillation can be observed in $A=A_{M}$ in LAD and $A=A_{FL}$ in LAD1 with all the input voltages are chosen in the limit cycles ranges. It is evident that the domain of spiking periodic oscillation could occur depending on the parameters of the proposed universal CCM model, which is not mentioned in the supercritical Hopf bifurcation theory. Finally, the non-periodic oscillation with a small amplitude could exist on the open LHP that that is far away from the Hopf bifurcation points in Figure 21d. In other words, the non-sinusoidal “spiking” periodic oscillation, periodic and non-periodic oscillation can coexist in the proposed universal model (1);

(3) Bursting oscillation

**Figure 22:**
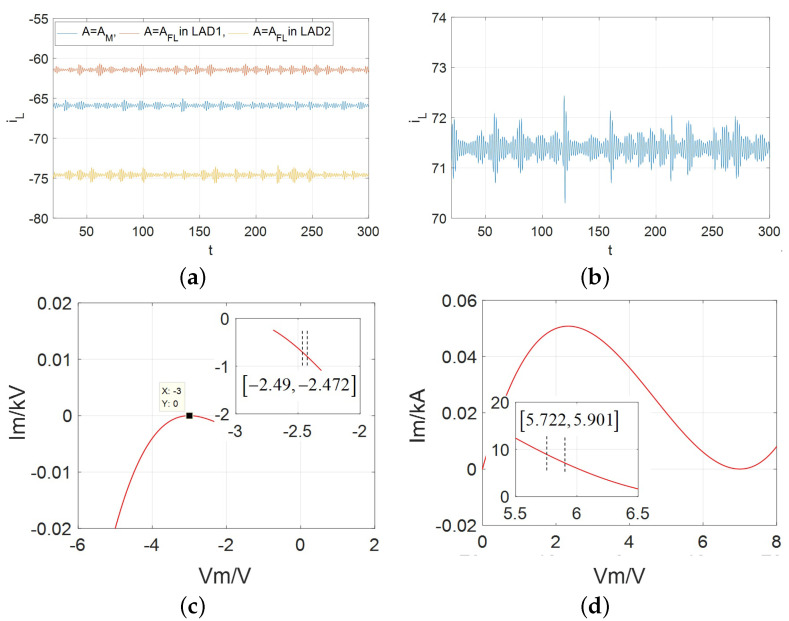
The bursting oscillation behaviors in the LAD for the proposed model: (**a**) at v=−2.485 V for $A=A_{M}$ over v∈[−2.49,−2.472] is graphed by blue solid line, at v=−3.82 V for $A=A_{FL}$ in LAD1 is graphed by green solid line, at v=−13.241 V for $A=A_{FL}$ in LAD2 is graphed by pink solid line; (**b**) at v= 5.9 V for $A=A_{FL}$ over v∈[5.722,5.901]; (**c**) The bursting region of $A=A_{M}$; (**d**) The bursting region $A=A_{FL}$.

From above figures, the distributions of the spiking oscillation (i.e., action potential) has been analyzed, which resemblance to biologically-generated action potentials at the same time, it verifies the Chua’s theory on “local activity” is the origin of action potential (spikes). In this subsection, in order to further observe the neural dynamics for the proposed CCM-based second-order circuit, the other significant nonlinear behavior is presented, such as bursting oscillation and their distributions, which are illustrated in Figure 22.

Observing all the bursting regions. They lie on the open LHP with a non-periodic small-amplitude oscillation, but unstable, and rapidly transition into a stable (oscillating) motion as *V* increases in LAD(s). Among them, the widest region is the case of $A=A_{FL}$, and the narrowest one is $A=A_{M}$. There are two regions that have been found in the case of $A=A_{FL}$. In other words, more complex nonlinearity and neural dynamics exist in the case of $A=A_{FL}$, such as edge of chaos, local active domain, the compensated positive inductance ($L^{*}$), limit cycle, supercritical Hopf bifurcation, spiking and bursting oscillation, and so on. As the essential of the bursting which implies a series of neural behaviors, which is one of the most important topics in the research of neuromorphic, such as biological adaptive behavior, long-short-term memory, biological emotion-/memory-like models, etc. Although all the bursting oscillations are distributed in a tiny-region(s), they still could be used to construct the emulated electronic neuron or neural network circuits. Clearly, the bursting phenomenon agrees with the engineering requirement in the field of biomimetic neurology, and is our primary contribution for the proposed CCM.

## 5. Conclusions

In order to gain in-depth understanding of the nonlinear characteristics of the Chua corsage memristor and explore the applications in the field of biomimetic neurology, the universal model and generation rules of the *m*-lobe Chua corsage memristor are introduced, as well as their natural characteristics. The novel small-signal equivalent circuit with one positive capacitance and two resistors is proposed and its impedance function is presented and analyzed to verify the related theory. Moreover, the zero-pole diagrams and frequency response of the admittance functions for the simplest oscillator are discussed in detail, according to the existence of the edge of chaos. Furthermore, the features and distribution of the compensated positive inductance ($L^{*}$) are discussed and summarized. In addition, the oscillation mechanism of the proposed CCM-based simplest oscillator is analyzed. Finally, the neural dynamics are demonstrated, such as the limit cycle, supercritical Hopf bifurcation, spiking and bursting oscillation, respectively. This study provided a theoretical foundation for application in the field of biomimetic neurology.

## Figures and Tables

**Table 1:** The relationship between parameters and the number of the lobes for the *m*-lobe CCM.

Parameters	N	m	a	$A=A_{M}$	$n_{j}=\left(j+1\right)\left(j+2\right),$ $h_{j}=n_{j}+1$, (j=1,2,3,…)	f(x,v)	Figure
2-lobe	1	2	0	9	$n_{1}=6$, $h_{1}=12$	9−x+|x−6|−|x−12|	Figure 3b
4-lobe	2	4	2	17	$n_{1}=6$, $h_{1}=12$, $n_{2}=20$, $h_{2}=30$	19−x+|x−6|−|x−12|+|x−20|−|x−30|	Figure 9a
6-lobe	3	6	5	28	$n_{1}=6$, $h_{1}=12$, $n_{2}=20$, $h_{2}=30$, $n_{3}=42$, $h_{3}=56$	33−x+|x−6|−|x−12|+|x−20|−|x−30|+|x−42|−|x−56|	Figure 9b
8-lobe	4	8	10	41	$n_{1}=6$, $h_{1}=12$, $n_{2}=20$, $h_{2}=30$, $n_{3}=42$, $h_{3}=56$, $n_{4}=72$, $h_{4}=90$	51−x+|x−6|−|x−12|+|x−20|−|x−30|+|x−42|−|x−56|+|x−72|−|x−90|	Figure 9c
10-lobe	5	10	17	57	$n_{1}=6$, $h_{1}=12$, $n_{2}=20$, $h_{2}=30$, $n_{3}=42$, $h_{3}=56$, $n_{4}=72$, $h_{4}=90$, $n_{5}=110$, $h_{5}=132$	74−x+|x−6|−|x−12|+|x−20|−|x−30|+|x−42|−|x−56|+|x−72|−|x−90|+|x−110|−|x−132|	Figure 9d
12-lobe	6	12	26	75	$n_{1}=6$, $h_{1}=12$, $n_{2}=20$, $h_{2}=30$, $n_{3}=42$, $h_{3}=56$, $n_{4}=72$, $h_{4}=90$, $n_{5}=110$, $h_{5}=132$, $n_{6}=156$, $h_{6}=182$	101−x+|x−6|−|x−12|+|x−20|−|x−30|+|x−42|−|x−56|+|x−72|−|x−90|+|x−110|−|x−132|+|x−156|−|x−182|	Figure 9e
14-lobe	7	14	37	97	$n_{1}=6$, $h_{1}=12$, $n_{2}=20$, $h_{2}=30$, $n_{3}=42$, $h_{3}=56$, $n_{4}=72$, $h_{4}=90$, $n_{5}=110$, $h_{5}=132$, $n_{6}=156$, $h_{6}=182$, $n_{7}=210$, $h_{7}=240$	134−x+|x−6|−|x−12|+|x−20|−|x−30|+|x−42|−|x−56|+|x−72|−|x−90|+|x−110|−|x−132|+|x−156|−|x−182|+|x−210|−|x−240|	Figure 9f
…	…	…	…	…	…	…	…

## Abbreviations

The following abbreviations are used in this manuscript:

MDPI	Multidisciplinary Digital Publishing Institute
DOAJ	Directory of open access journals
CCM	the Chua corsage memristor
$G_{0}$	a real constant
A	one real parameter
$A_{M}$	the midpoint
$A_{FS}$	the first segment
$A_{FL}$	the last segment
$Q_{m}$	the equilibriums (*m* = 0, 1, 2, …)
$T_{m}$	the turning points (*m* = 0, 1, 2, …)
V	the voltage
I	the current
$V_{m}$	the voltage of the proposed memristor
$I_{m}$	the current of the proposed memristor
$R_{m}$ and $R_{n}$	the resistance of two resistors
$C_{m}$	the capacitance of one capacitor
POP	the power-off-plot
DRM	the dynamic route maps
LAD	the local active domain
RHP and LHP	right-half plane and left-half plane
Y and Z	the equivalent asmittance and impedance
